# Birth and death of gene overlaps in vertebrates

**DOI:** 10.1186/1471-2148-7-193

**Published:** 2007-10-16

**Authors:** Izabela Makałowska, Chiao-Feng Lin, Krisitina Hernandez

**Affiliations:** 1 The Huck Institutes of the Life Sciences, The Pennsylvania State University, University Park, PA 16802, USA; 2 Institute of Molecular Evolutionary Genetics and Department of Biology, The Pennsylvania State University, University Park, PA 16802, USA; 3 Institute of Bioinformatics, University of Muenster, 48149 Muenster, Germany

## Abstract

**Background:**

Between five and fourteen per cent of genes in the vertebrate genomes do overlap sharing some intronic and/or exonic sequence. It was observed that majority of these overlaps are not conserved among vertebrate lineages. Although several mechanisms have been proposed to explain gene overlap origination the evolutionary basis of these phenomenon are still not well understood. Here, we present results of the comparative analysis of several vertebrate genomes. The purpose of this study was to examine overlapping genes in the context of their evolution and mechanisms leading to their origin.

**Results:**

Based on the presence and arrangement of human overlapping genes orthologs in rodent and fish genomes we developed 15 theoretical scenarios of overlapping genes evolution. Analysis of these theoretical scenarios and close examination of genomic sequences revealed new mechanisms leading to the overlaps evolution and confirmed that many of the vertebrate gene overlaps are not conserved. This study also demonstrates that repetitive elements contribute to the overlapping genes origination and, for the first time, that evolutionary events could lead to the loss of an ancient overlap.

**Conclusion:**

Birth as well as most probably death of gene overlaps occurred over the entire time of vertebrate evolution and there wasn't any rapid origin or 'big bang' in the course of overlapping genes evolution. The major forces in the gene overlaps origination are transposition and exaptation. Our results also imply that origin of overlapping genes is not an issue of saving space and contracting genomes size.

## Background

3.2 billion base pairs of the human genome harbor about 23,000 protein coding genes. With the average size of a gene equal to 48 kb, they cover approximately one third of the genome. It seems that there's enough space in the genome for each gene to be separated by a large distance. Yet, between five and fourteen per cent of genes in the vertebrate genomes do overlap [1]. The unexpected abundance of complementary pairs of sense/antisense transcripts poses major challenges to achieve a comprehensive understanding of a gene structure and expression at the genomic level. Studies of individual overlapping gene pairs in eukaryotes have shown that they regulate gene expression by different mechanisms such as genomic imprinting [2], RNA interference and translational regulation [3], transcriptional interference [4], alternative splicing [5], and X-inactivation [6]. Many genes involved in overlaps are known to be involved in disease development, e.g. *CACP *gene is responsible for camptodactyly-arthropathy-coxa vara-pericarditis syndrome [7] or are responsible for some important morphological features, e.g. *SLC24A5 *is partially responsible for skin coloration [8].

Several mechanisms have been proposed to explain gene overlap origination. For instance, Keese and Gibbs [9] suggested that overlapping genes arise as a result of overprinting – a process of generating new genes from preexisting nucleotide sequences. This process supposedly took place after divergence of mammals from birds and overlapping genes represent young, phylogeneticaly restricted genes encoding proteins with diverse functions, and are therefore specialized to the present life-style of the organism in which they are found. Shintani et al. [10] suggested that the overlap between genes *ACAT2 *(acetyl-Coenzyme A acetyltransferase 2) and *TCP1 *(t-complex 1) arose during the transition from therapsid reptiles to mammals in one of two ways. In one scenario, one of genes was translocated and the rearrangement has been accompanied by the loss of a part of the 3' UTR, including the polyadenylation signal, from one gene. By chance the 3' UTR of the new neighbor on the opposite strand contained all the signals necessary for transcription termination so that the translocated gene could continue to function. Alternatively, the two genes become neighbors through the rearrangement but at first did not overlap. Later, one of the genes lost its original polyadenylation signal, but was able to use a signal that happened to be present on the non-coding strand of the other gene. The *ACAT2-TCP1 *overlap evolution was placed, similarily as in Keese and Gibbs hypothesis, after the divergence of mammals from birds. Dahary et al. [11] place the origin of most vertebrate overlaps much earlier. They found that human antisense genes have largely conserved linkage in torafugu which may imply that big fraction of human overlapping genes represents vertebrates' ancestral overlaps. However, our previous study of human and mouse overlapping genes showed that even between closely related species overlaps are not that well conserved [12]. Out of 255 cases in which both members of the human overlapping gene pair had mouse orthologs, only 95 were overlapping in both species. In addition, significant fraction of these 95 gene pairs show different overlap patterns in the two genomes. Lack of the overlap conservation was also observed in other studies [13-15].

Here we present results of the comparative analysis of seven vertebrate genomes: human, chimpanzee, mouse, rat, chicken, fugu, and zebrafish. This comparative study shows that on one hand, many of the vertebrate gene overlaps are not conserved and are lineage specific. On the other hand, this work reveals new, not published before, cases of genes overlap conservation in vertebrates. We also show new mechanisms of overlapping genes evolution and demonstrate, for the first time, that evolutionary events could not only lead to the new gene overlaps origin but also to the loss of an ancient overlap. Therefore lack of strong overlaps conservation between even closely related species may result from the origin of a new, lineage specific overlap as well as from the loss of overlaps in many lineages. Findings about evolutionary changes in the gene structure and organization are very important in our quest toward understanding genomes and genes expression. Changes in the gene structures may lead to modifications in the gene expression and expression correlation between involved genes, which may further explain some differences between species such as discrepancies in the orthologous genes expression patterns [16].

## Results

### Conservation of overlaps in vertebrate genomes

Fraction of the overlapping genes in various vertebrates differs significantly (Table 1). In tetrapoda over 10% of all genes are involved in some type of overlap, while in fish only 5–7%. The exception is the rat genome where only 4.87% of all genes are overlapping with another gene. However, this is most likely due to the annotation incompleteness and not a specific feature of the rat genome. Many rat genes do not have UTRs annotated and as we learned from this and other studies [12,17,18] the majority of gene overlaps are in the UTR regions. Incomplete annotations are likely to be responsible for some discrepancies between human and chimpanzee and could also be true for fish genomes. Another possibility is that many overlaps evolved after *Actinopterigii *diverged from *Sacropterigii *and most overlaps observed in these lineages arose independently. Differences in overlapping genes frequencies between human and other species were tested using chi-square test (Table 1). In all cases but mouse the chi-square is higher than critical value at α = 0.0005 and therefore differences are statistically significant. For mouse the difference is significant at α = 0.05.

**Table 1:** Overlapping genes in vertebrate genomes

	Number of genes analyzed	Number and fraction (in parentheses) of genes involved in overlaps	Chi-square test value (when compared to human)	Number of overlaps	Nested genes	Exon/exon overlaps (NATs) *		Intron/exon overlaps*
							
						Total	CDS involved	
Human	22,291	2,978 (13.4)	NA	1,766	972	634	417	160
Chimpanzee	21,506	2,219 (10.3)	73.0888	1,276	665	479	317	132
Mouse	25,383	3,456 (13.6)	5.0750	2,053	1,071	819	565	163
Rat	22,159	1,080 (4.9)	895.1585	607	458	102	100	47
Chicken	17,709	1,960 (11.1)	32.2585	1,135	474	511	471	150
Fugu	20,796	993 (4.8)	880.5199	556	174	290	290	92
Zebrafish	23,524	1,625 (6.9)	472.4534	1,026	767	98	85	161

* excluding nested genes

Number of genes involved in overlaps is smaller than the number of overlaps multiplied by two since some genes are involved in more than one overlap. Multiple genes may be nested in one host gene as well as a gene may be overlapping other genes on both ends as reported previously [12]. Differences in overlapping genes frequencies between human and other species were tested using chi-square test. In all cases but mouse the chi-square is higher than critical value at α = 0.0005 and therefore differences are statistically significant. For mouse the difference is significant at α = 0.05. However, no definite conclusions can be drawn by this test results since some differences may result from the annotation problems but not real differences in the overlapping genes fraction.

Table 2 shows results of the analysis of overlap conservation, which indicate that sets of overlapping genes differ between species. This demonstrates that many overlaps are species or lineage specific and only a small fraction of them are shared among vertebrates. Although some conserved overlaps are not observed due to missing data, we can assume that this would affect both, species specific and conserved overlaps in similar way and therefore the proportions and the general picture are not affected. Similar disproportion in the sense-antisense transcripts abundance was also shown by Zhang et al. [19].

**Table 2:** Overlapping genes conserved between species

	Human	Chimpanzee	Mouse	Rat	Chicken	Fugu	Zebrafish
Human	-	1100	274	98	64	23	17
Chimpanzee	477	-	NA	NA	NA	NA	NA
Mouse	146	NA	-	141	76	26	16
Rat	11	NA	48	-	45	19	6
Chicken	9	NA	10	2	-	22	13
Fugu	1	NA	0	0	0	-	13
Zebrafish	5	NA	5	0	0	1	-

Above diagonal shows total number of conserved overlaps, and below diagonal shows numbers of conserved exon/exon overlaps. For chimpanzee the data is provided only in relation to human, gene orthology relation between chimpanzee and other than human species is not established and annotated in the Ensemble database yet.

### Patterns of human overlapping genes evolution

Using Ensembl gene homology data [20] we identified homologs of human overlapping genes in other species. Out of 2,978 human genes involved in overlaps, 264 were human specific and had no homologs in any other analyzed genome, including chimpanzee. Interestingly, we couldn't find a rodent homolog for about 25% of human overlapping genes, whereas genome wide comparison shows that 89–90% of rat genes possess a single ortholog in the human genome [21]. Similarly, it was observed that 25% of human genes do not have torafugu orthologs [22], while our study shows that in the case of the overlapping genes 46.17% of human genes lack a torafugu ortholog. These results imply that a lot of genes involved in overlaps are young, lineage specific genes and do not have orthologs in other lineages. This supports the 'overprinting' hypothesis but is in sharp contrast to observation made by Dahary et al. who based on comparison of the human and torafugu genomes concluded that most human overlaps are ancient [11]. However, their conclusion may be an artifact of the applied method, because they analyzed only those human genes that have identifiable orthologs in the torafugu genome.

Based on the presence or absence of an ortholog in species representing two other lineages, i.e. rodents and fish, we divided human overlapping genes pairs into those that have: both orthologs in both lineages (476 pairs); both orthologs in rodents and only one in fish (279 pairs); both orthologs in rodents and none in fish (111 gene pairs); an ortholog of one gene only in both lineages (466 pairs); ortholog of one gene in rodents and none in fish (200 pairs); and no orthologs in neither lineage (92 pairs). Next, we analyzed genomic arrangement in all cases where both orthologs of human overlapping genes were found. According to our results we divided gene pairs into: overlapping (if they also overlap in particular species), neighboring (if they were not overlapping but placed one next to each other without any gene between them), and separated (if they were on different chromosomes, contigs or were separated by other genes).

**Figure 1:**
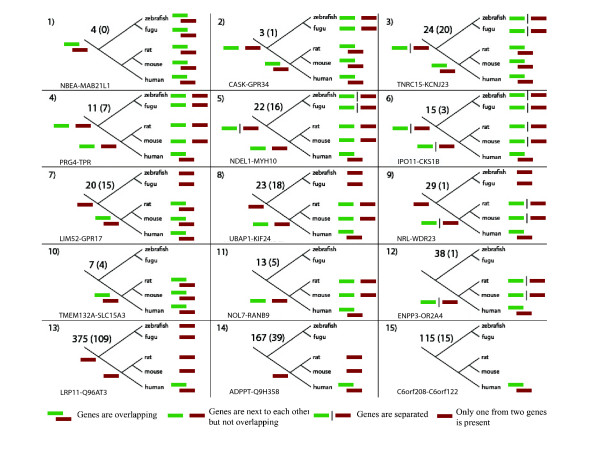
Putative patterns of human overlapping genes evolution together with examples from our data set. Numbers in parenthesis represent cases of exon/exon overlaps in each category. In each case an example from our studies is given. Analysis was done based on the October 2004 Ensembl release. The bar between two genes indicates that the genes are located on different chromosomes.

Considering previously published major events leading to the gene overlaps; genes rearrangements or transposition, extension of genes by adoption of signals or new exons, and new genes origination [9,10] we developed 15 theoretical scenarios of overlapping genes evolution (Figure 1). Results from the above analysis provide support for every theoretical pattern of overlap evolution. Presented examples, for each scenario, were carefully examined and confirmed by the presence of cDNA and/or EST sequences. Also, sequence analysis was performed to ensure the missing orthologs are truly not present in a given species or lack of overlaps is not resulting from annotations problem. Our requirements here were very conservative and each gene pair where there was disagreement between species from the same lineage was removed from studies.

### Mechanisms leading to gene overlaps

Gene overlaps evolve by a variety of mechanisms and not by a single universal mechanism. Essentially, any mechanism that gives rise to a new gene, such as gene duplication or retroposition, may result in a gene overlap. Alternative splicing represents another major source of proteome diversity in mammals and origination of a new splice form may lead to a gene overlap as well.

In the analyzed data, we found cases supporting all the proposed hypotheses of gene overlap origination, i.e. overprinting, a gene translocation, or adoption of a new transcription termination signal (changing a gene structure in more general terms). Among identified human overlapping genes in 115 cases both genes involved in the overlap did not have the ortholog in neither rodent nor fish lineage, in 64 cases one ortholog was present in rodents and none in fish, and only in 68 cases both orthologs were present in rodent and fish genomes. Figure 1 shows fifteen scenarios of overlapping gene evolution. Patterns 7, 13, and 14 (Figure 1) fit exactly the overprinting hypothesis because they show human overlaps where one of gene is an old gene present in all vertebrates and the second gene is a young one not present in fish nor rodents. Dan et al. [13] showed that a recently evolved overlap between MINK and CHRNE genes resulted from mutations in the polyadenlylation signal and acquisition of a new downstream signal within a neighboring locus. Evolutionary scenarios represented by models 2–6, 8, 9, 11, and 12 (Figure 1) indicate involvement of translocation and possible signal adoption in the overlapping genes origin.

Although our models support published hypotheses we should consider much broader range of events which could lead to genes overlaps. Summarizing published hypotheses in a more general way we can say that major events playing a role in overlapping genes evolution are: translocation (or transposition), change in the gene structure (extension of UTR would fall into this category), and development of a new gene or a new splice variant.

#### Development of a new splice variant

Gene overlaps might not be conserved among species due to different gene structures [12]. In addition to adopting a new termination and an extension of the last exon, conversion of the previously unused genetic material in the form of a new splicing variant may lead to the gene overlap. There are two possible scenarios, an additional splice variant arises or the ancestral variant may be replaced by a new one.

Developing a new, additional, splice variant may be considered as a special case of overprinting since the new splice variant represents a new transcript. In fact, the case described by Keese and Gibbs [9] falls into this category because reported the new gene is just a new splice variant of TRalpha (TRHA) gene. Comparative analysis of the genomic region containing TRHA and NR1D1 (nuclear receptor subfamily 1, group D, member 1) genes revealed that the overlap is conserved among placental mammals, who have two splice variants of TRHA. Only one of these, the one which does not overlap with NR1D1, was identified in marsupials and all non-mammalian lineages. Close examination of the genomic region alignments showed that an insertion of new genetic material occurred some time after divergence of placental mammals and this inserted sequence was used for a new splice variant. This finding disagrees with the overprinting hypothesis as a new variant wasn't built from old existing material but rather new genetic information, not present in other genomes. However, we can not exclude possibility that this genomic fragment wasn't lost in other genomes.

**Figure 2:**
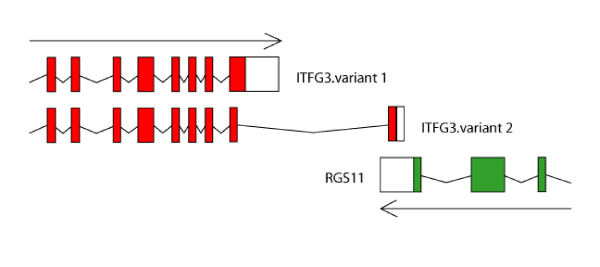
Genomic organization of ITFG3 and RGS11 in primates. In other mammals, although, these genes are neighbors on the same chromosome, variant 2 of ITGG3 is not observed and genes do not overlap. Coding sequences are colored and empty boxes denote 3'UTRs of both genes. 5' end of both genes were trimmed for the presentation purpose.

The same mechanism can be attributed to the origination of ITFG3 (integrin alpha FG-GAP repeat containing 3) and RGS11 (regulator of G-protein signalling 11) overlap in primates. ITFG3 has two splice variants in primates and one of them is overlapping at 3' end with RGS11 gene. Figure 2 shows genomic organization of both genes in human; similar organization is observed in the chimpanzee and macaque genomes. In all non-primate species only the non-overlapping (shorter) variant is present. To exclude the possibility that the overlapping splice variant was missed in annotations in non primate genomes we investigated alignments of human and other genomes in two genomic browsers, Ensemble and UCSC browser. In both cases there was not a good alignment in the region occupied by the primate specific exon. Similarly, search against GenBank databases did not reveal any similarity between proteins encoded by the overlapping exon and any other non primate protein, genomic or EST sequence. This clearly shows that overlapping splice variant of gene encoding ITFG3 is lineage specific and arose recently after divergence of primates.

Another example of primate specific overlap that resulted from a new splice variant is a pair of genes THAP3, THAP domain containing, apoptosis associated protein 3, and DNAJC11, DnaJ (Hsp40) homolog, subfamily C, member 11. Both THAP3 and DNAJC11 homologs were found in a majority of analyzed vertebrate species: human, macaque, chimpanzee, mouse, rat, dog, cow, opossum, and zebrafish. Interestingly, the THAP3 was missing in chicken, frog, and tetraodon. We couldn't identify the gene in these genomes by any standard comparative methods including BLASTn and tBLASTn. However, zebrafish is apparently not the only fish species THAP3 gene. EST sequences DT157701, DT154094, DT175180, DT175179, and DT157700 from *Pimephales promelas *show high similarity to zebrafish THAP3 protein (64–77% identity) and likely represent THAP3 transcript. In primates, THAP3 has two splice variants one of which overlaps with DNAJC11. In all other species only one, shorter variant is present and it is not overlapping with DNAJC11. Comparative analysis of genomic sequences in the region of the overlap shows that there is no conservation in this region and most likely the longer variant is primate specific. We also did not identify any EST sequence which could show the presence of longer (overlapping) variant in non primate vertebrates. This analysis led to conclusion that longer splice variant of THAP3 and THAP3-DNAJC11 overlap are primate specific.

#### Development of a new gene

The new splice variant origination seems to be one of the most common events leading to the lineage specific overlaps. However, our data strongly suggest that this is not the only case where we observe 'overprinting'. The powerful evidence that origination of new lineage specific genes plays a big role in the evolution of overlapping genes comes from data that many human genes do not have orthologs in other lineages. It is known that the many of human genes are not found in rodent [21], chicken [23] or fish [22] genomes, so our result could just reflect these findings. Interestingly, the fraction of human genes with missing orthologs is higher for overlapping genes than non-overlapping genes. Approximately 10% of human genes are missing in mouse genome while 27% of human overlapping genes are missing in mouse. Similarly, about 20% and 24% of human genes are not found in chicken and torafugu but this fraction is much higher in our studies: 44.9% and 46.17% in chicken and torafugu, respectively.

**Figure 3:**
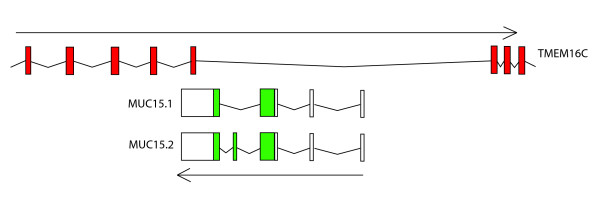
Genomic organization of TMEM16C and MUC15 in mammals. In chicken, Xenopus and zebrafish MUC15 is not observed.

A confirmation for new gene origination as a source of gene overlap comes from the case of TMEM16C, transmembrane protein 16C, and MUC15, mucin 15; located on 11p14.2-3. Human TMEM16C has 27 exons spanning 331,865 base pairs. Two splice variants of MUC15 are embedded in the TMEM16C gene occupying introns 13 and 14 and overlapping, in the 3'UTR area, with exon 14 (Figure 3). Gene MUC15 is present only in mammalian genomes and there is experimental evidence that it is overlapping with TMEM16C at least in primates, rodents and cow. This gene is not present in any other lineages including chicken, xenopus and zebrafish and alignment of genomic sequences shows no conservation in areas covered by MUC15. However alignments at the protein level show some traces of similarity in chicken in MUC15 exon four of splice variant 2 and in xenopus in the same exon four in part of exon three. We could not detect any similarity in zebrafish as well as in invertebrates. The MUC15 sequence appears to be specific for vertebrates only and there are two possible evolutionary scenarios. In one, the sequence was present in early chordates and in the process of neutral mutation gained the coding potential which was used to build a new gene in mammalian ancestor. Another possibility is that this gene, was lost in majority of lineages with traces left in few genomes, and was maintained only in mammals. At any rate TMEM16C and MUC15 represent an overlap between an old gene TMEM16C and a newer mammalian specific gene (MUC15).

#### Changes in the gene structure

In the cases described above the gene overlap evolved through the origin of a new, longer splice variant. In many instances we observed a slightly different situation, a new variant arose and replaced the ancient one, so the number of variants was the same in analyzed lineages; however, they differ in their genomic organization. Examples of ACAT2-TCP1 [10] and MINK-CHRNE [13] overlaps are simple cases of changes in the gene structure where the most 3' exon was extended as a result of adopting the closest polyA signal after the original one was lost.

**Figure 4:**
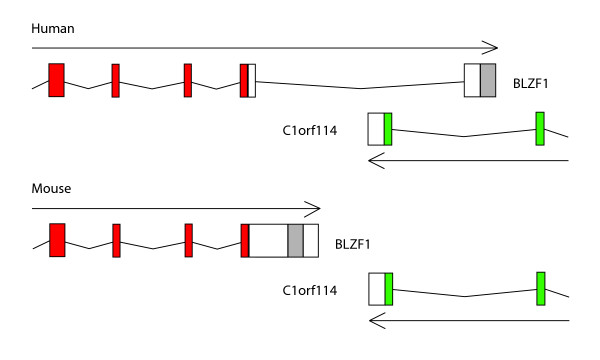
Genomic organization of human and mouse BLZF1 and C1orf114 genes. Shaded areas of 3'UTRs represent repetitive elements location.

Example of BLZF1 gene (basic leucine zipper nuclear factor 1), overlapping at 3' end with the gene C1orf114 (open reading frame 114 on human chromosome 1, position 167603818–167663296) shows a more drastic shift in the gene structure. This overlap exists in the human and chimpanzee genomes but the two genes are neighbors, and do not overlap in other mammals including opossum where they are located on chromosome 2 about 6 kb apart. A similar arrangement is observed in chicken (chromosome 1, 2 kb separation) and in Xenopus (the same scaffold, 9 kb separation). Interestingly, in zebrafish these two genes are located on different chromosomes – BLZF1 on chromosome 1 and a homolog of C1orf114 most likely is located on chromosome 8. Although there's no gene annotated in the cognate region, both human protein similarity and zebrafish EST alignments strongly suggest existence of the C1orf114 gene in this region. Figure 4 shows genomic organization of these overlapping genes in human and mouse. Analysis of multiple alignments of the region containing the last exon of BLZF1 in human and corresponding region in other vertebrates revealed that this fragment is not conserved among vertebrate lineages. Clearly there was an insertion in an ancient primate genome; a fragment belonging to the last, primate specific, exon does not align with non-primate genomes. Analysis of this fragment showed that the very 3' end of human BLZF1 is occupied by AluS, an old primate specific retroelement. Similarly, middle part of the last BLZF1 exon in mouse, contains rodent specific element B4A. Apparently independent insertions of repetitive elements occurred in both, primate and rodent, genomes and both are associated with exonification and new splice variant development.

#### Gene duplication and retrotransposition

Gene duplication is a common mechanism for the origin of new genes [24,25]. Retrotransposition is an interesting mechanism that allows a gene to move to a distant location on the same or different chromosome. Retro(pseudo)genes are products of reverse transcription of a spliced (mature) mRNA and they are characterized by lack of introns, presence of polyA track, and flanking direct repeats. Because they are copies of mature mRNAs, they usually lack promoters and cannot be transcribed. However, in some rare instances, after insertion near an existing promoter or exaptation of anonymous sequence as a promoter, they can gain transcriptional activity and create a new functional gene [24,26].

An example of a new gene overlap due to formation of a new gene origination comes from the ribosomal protein RPS27 retrogene and TSPAN9 (tetraspanin 9, known also as NET-5) gene. RPS27 has two intron-containing paralogs: RPS27 and RPS27L, and both of them gave rise to multiple retrocopies in the human genome. We identified 24 retro(pseudo)genes of RPS27; ten of them are nested in another gene. Although multiple RPS27 retrogenes can be identified in other mammalian genomes, none of the host-nested gene pairs are the same in the human and rodent genomes. The aforementioned retrocopy of RPS27, nested in the human tetraspanin 9 gene, has an intact open reading frame and potentially encodes for 84 amino acid protein 100% identical to the spliced version of the gene on chromosome 1. We also identified two EST sequences, AV763564 and CD386048 that are 99% identical to this gene and show weaker similarity to other RPS27 genes, which may imply that this gene is expressed. However, because of relatively low quality of EST sequences, these results are not conclusive and further analysis would be required to confirm expression of this gene. This retrosequence is present in the human and chimp genomes but missing from orthologous location in macaque and all other vertebrates that we analyzed. This confirms recent origin of the RPS27 retrosequence and makes its expression assessment based on an intact ORF impossible. However, this example demonstrates a potential route to new overlaps in the vertebrate genomes.

### Loss of gene overlaps

**Figure 5:**
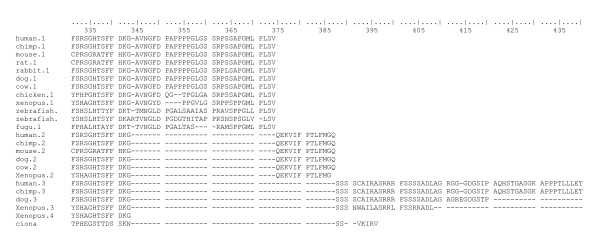
Alignments of vertebrate NDEL1 proteins coded by all splice variants.

**Figure 6:**
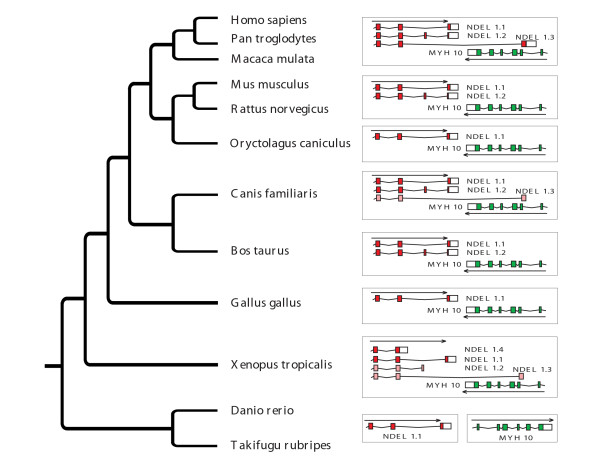
Phylogenetic tree with MYH10 and NDEL1 variants and genomic organization in vertebrates.

While young gene overlaps can arise from new splice variants, ancient overlaps can disappear due to the loss of the overlapping splice variant. In fact, we did observe such cases in our dataset that showed that analysis of narrow range of vertebrate or mammalian lineages may lead to false conclusions due to incompleteness of the data. The human NDEL1 gene coding for a thiol-activated peptidase and MYH10 gene that codes for myosin heavy chain 10 reside on chromosome 17 and they overlap in primates but not in rodents, chicken or fish. In primates there are three splice variants of NDEL1 and only one is overlapping with MYH10. In mouse and rat only two non overlapping transcripts are present, and in chicken we observe only one variant. The same variant as in chicken is present in zebrafish, however NDEL1 and MYH10 genes are separated. In addition, in zebrafish NDEL1 has two copies representing the same splice variant, one located at chromosome 12, position 35 35,318,946–35,338,461 and another located at chromosome 3 at position 56,442,046–56,469,437. MYH10 in zebrafish is on chromosome 6, position 5,082,544–5,106,092. Based on these observations the most parsimonious explanation would be that one of these genes was translocated after fish divergence and later on the overlapping splice variant arose in primates. However, when we looked at the genomic arrangement of these genes in other species we observed that the dog genome has all three primate's splice variants. Further analysis showed that *Xenopus *has even more, four splice variants, two of them confirmed by cDNA and EST sequences and two predicted. One of the predicted variants is overlapping with MYH10 and protein coded by this variant shows high similarity to protein encoded by overlapping transcript observed in primates and in dog. Analysis of all known vertebrate transcripts and proteins encoded by them suggests that in a tetrapoda ancestor genome there were at least three splice variants of NDEL1. During the evolution one or two of them were lost in most lineages. Only primates retained three variants and all of them are confirmed by EST or cDNA sequences. In dog and *Xenopus *overlapping variant was predicted but it is shorter than the one in primates and up to date there is no experimental confirmation for its expression. However, the analysis of proteins coded by these variants (Figure 5) clearly shows that the overlapping splice variant in *Xenopus *is the same as the one in dogs and primates. In zebrafish both copies of NDEL1 represent variant 1 that seems to be the most common in vertebrate genomes. Variants of NDEL1 most probably evolved at early chordates because only one is present in *Ciona intestinalis*. Sequence divergence did not allow us to establish which, if any, of three variants is shared with *Ciona intestinalis*. Figure 6 shows the phylogenetic tree with all analyzed species. Bars to the right represent splice variants of NDEL1. The most likely mechanism of the variant loss in some lineages is a weakening of the splicing signal that leads to skipping of an exon. Similar effect can be observed in case when the other exon acquires signal so strong that dominates completely splicing and results in a constitutive exon. Another possibility is that this transcript is using signals from L1 elements which are in its 3' UTR. [27] These elements are in human sequence but are partially or completely lost in other genomes. However, more extensive analyses that would include some wet lab experiments are required in order to determine what the case is here.

### Time of overlapping genes evolution

**Figure 7:**
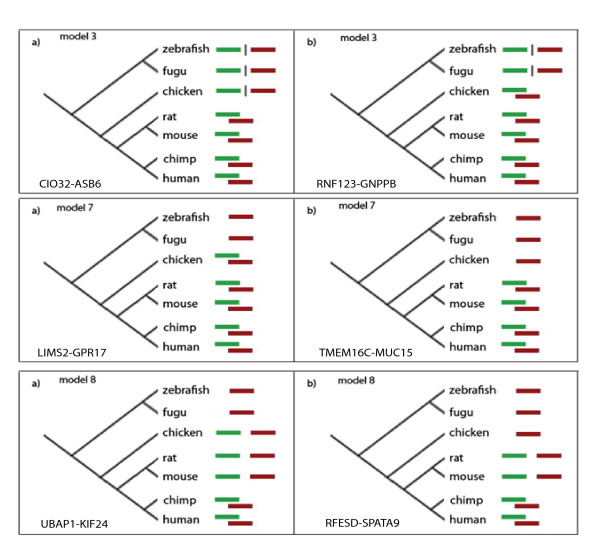
Examples of genomic arrangements of human overlapping genes orthologs in chicken. In left column genes arrangement in chicken resembles this in primates and rodents, in the right column genes in chicken are arranged the same way as in fish.

Equally important, to the mechanism leading to overlaps, is the time when particular event occurred. Two widely accepted hypotheses of vertebrate overlapping genes evolution [9,10] assume that explosion of gene overlaps occurred in early mammals. On the contrary, Dahary et al. [11] and Zhang et al. [19] suggested that most naturally occurring anti-transcripts observed in human represent ancient vertebrate gene overlaps. To answer this question, we looked for evidence of putative gene overlap before and after mammalian radiation. We studied cases where the pattern of gene arrangement differs in fish and rodent, i.e. two genes overlap in rodents but not in fish. As shown in Figure 7, genes arrangement in the chicken genome in some cases is similar to the one in rodents in other to the one in fish. For example genes CIO32 and ASB6 overlap in mammals only but genes RNF123 and GNPPB overlap also in chicken. In another example gene pair UBAP1 and KIF24 is overlapping in human but not overlapping, although located next to each other, in rodents and chicken. Only one of these genes, UBAP1 is present in fish. On the other hand, genes RFESD and SPATA9 overlap in human but not in rodents where they are located next to each other. In chicken, as well as in fish gene SPATA9 is not found. This demonstrates that the events leading to gene overlaps happened before and after mammalian radiation. The development of gene overlaps is a long, continuous process, not a 'big bang' that happened during a specific short period of vertebrate evolution. Also examples discussed in previous sections showed that gene overlaps were evolving at different stages of vertebrate evolution and could arise in early chordates, early mammals, or just recently in primates and other mammalian lineages.

## Discussion

Although large scale studies of overlapping genes have been available since 2002 we still do not understand how these overlaps evolved and what, if any, is the functional meaning of sharing the genomic locus between genes in eukaryotic genomes. Results published so far show evidence for both, relatively new, lineage specific [9,10,13] as well as conserved overlaps among vertebrate [11,19] and even all eukaryotes [28] gene overlaps. However, none of the papers, even those with gene overlaps origin hypotheses, fully explains this evolutionary phenomenon. This study brings us a little closer to that goal and the major conclusion is that there's no single mechanism responsible for the overlap origination. In principle, any mechanism of a new exon or a new gene origination may lead to a gene overlap. In the light of presented results, we can conclude that the major forces in the overlapping genes evolution are transposition and exaptation – a process that gives rise to new genes or new variants from preexisting nucleotide sequences. [29]. UTR extension in the course of new polyA signal adoption [10,13] or new splice variant development [9] are perfect examples of exaptation. Another type of exaptation is building a new gene structure by adopting an inserted transposable element. Transposable elements are known to contribute to host gene regulation [30,31] or structure [32,33]. Our study on BLZF1 and C1orf114 showed that transposable elements also contribute to origin of a new class of genetic novelties, namely overlapping genes. The analyzed data also provided evidence that new gene origination is truly observed in the process of overlaps evolution supporting even further hypothesis by Keese and Gibbs [9]. 'Overprinting' hypothesis was constructed based on the new splice variant origination. Overlap between TMEM16C and mammalian specific gene MUC15 showed that overlaps may involve pairs of an old ancient gene and a new lineage specific gene. However, we cannot agree that this is true for all vertebrate overlaps as hypothesized. In many of analyzed gene pairs both genes were old and conserved through eukaryotes.

Nevertheless, our study shows a number of gene overlaps that are lineage specific and are not conserved among vertebrates which supports our earlier studies of overlaps in human and mouse [12]. This is in a contradiction with the study of Dahary et al. [11] on human and fugu genomes that concludes that most human overlaps are ancient. However, they analyzed only those human genes that have identifiable orthologs in the fugu genome and therefore, young overlaps involving lineage specific genes were excluded from the study by definition. Also, their judgment was based on cases of overlapping human genes that were on average closer to each other in the fugu genome then other genes, and not based on true conserved overlaps. So, some of the gene pairs in fugu although close one to each other, are not necessary overlapping as is clear from our analysis.

In summary, we should emphasize that overlapping genes do not present any special case in regard to mechanisms of evolution. Events like gene translocation or exaptation, driving forces in genome evolution, are also common and major mechanisms in gene overlaps origin. There wasn't also any rapid origin or a 'big bang' of the overlapping genes after the split of bird and mammal lineages as suggested by Keese and Gibbs [9], nor are most of the human overlaps ancient as described by Dahary et al [11]. Birth as well as most likely death of gene overlaps is a continue process that occurred over the entire time of vertebrate evolution, similarly like any other genes arose or die over a long process of the eukaryotic genomes evolution [34].

Our results also imply that origin of overlapping genes is not an issue of saving space and contracting genomes size. Although there are some implications on functional importance of overlapping genes, the present analysis shows that most gene overlaps evolve stochastically, the same way as other genomic features, and without any positive pressure on the overlap presence. If overlaps have some functional meaning it is not a common case and most likely this function evolved by chance as a consequence of new genes arrangement.

This study also demonstrates that in order to fully understand the evolution of overlapping genes one has to study many genomes in minute details. Studies on a limited number of species may lead to false conclusions as shown in the case of NDEL1 and in many other cases we investigated during this study. Many gene pairs were moved from one category to another as a result of detailed examination of annotations and additional analysis. This shows that although human and other genomes are considered to be complete, their annotation is still far from final and in many cases cannot be trusted. Therefore, careful examination of any gene pair by a human expert followed by, in an ideal world, some wet-lab experiments is a key to sound results. We are very well aware that the present study did not solve all the questions regarding overlapping genes evolution and their origins. However, it did shed a light on how some of these overlaps evolved, provided a strong confirmation for lineage specific overlaps, and delivered firsthand evidence of gene overlap loss in the vertebrate lineage.

## Methods

### Sequence data

Assembled sequences and annotations of seven analyzed genomes were downloaded from Ensembl [20] and stored in a local mySQL database. We used following versions of the genomes: human-24.34e (NCBI 34), chimp-24.1 (CHIMP 1), mouse-24.33 (NCBI m33), rat-24.3c (RGSC 3.1), chicken-24.1a (WASHUC 1), fugu-24.2c (FUGU 2.0), and zebrafish-24.4 (Zv 4).

### Identification of the overlapping genes

For practical reasons, we applied an operational definition of a gene, as a part of the genomic region from the beginning to the end of an annotated transcript. Any two genes, defined as above, whose coordinates overlap and are transcribed from the different DNA strand, are considered as overlapping.

### Identification of orthologous genes and mapping information

Orthology inference was done based on any two genomes homology information provided in Ensembl. The set of overlapping genes for a given species was always a starting point for each orthology analysis. As a result seven by seven orthology matrix was created. It is important to stress that orthology relationship provided by Ensembl is not a simple one-to-one relationship. Whenever lineage specific gene duplication is detected one-to-many orthologs are provided. In these cases, each of several orthologs was checked for the overlaps. The detailed description of the method is available at Ensembl webpage [35]. Additionally, we used conserved synteny information of the neighboring genes to enhance reliability of the orthology inference. However, not all the genes have had their orthologs listed. In these cases, we assumed that a cognate gene is missing from a given genome.

The mapping information of each orthologous gene was downloaded from the Ensemble. For each pair of overlapping genes in one genome, e.g. human, spatial relationship of their orthologs in the other six genomes was checked based on existing annotation.

### Extending neighboring but not overlapping genes

We mapped TIGR gene indices [36] to all neighboring but not overlapping orthologs of human overlapping genes to check for possible extensions. In each case, we extracted genomic fragments containing a pair of neighboring genes and BLAST [37] against corresponding TGI sequences. Next we mapped transcripts to genomic fragments together with TGI sequences obtained from BLAST search. Only sequences showing similarity over 98% and fully aligning with the genomic fragment were used in order to avoid false positive hits to repetitive elements and ESTs from related genes. Results were stored in ASN.1 format and examined in Sequin [38].

### Multiple alignments

MultiZ alignments of genomic sequences were obtained from UCSC genome browser [39]. Protein multiple alignments were constructed using Clustalw [40].
